# *De novo* whole-genome assembly and resequencing resources for the roan (*Hippotragus equinus*), an iconic African antelope

**DOI:** 10.1093/g3journal/jkab002

**Published:** 2021-01-23

**Authors:** Margarida Gonçalves, Hans R Siegismund, Bettine Jansen van Vuuren, Klaus-Peter Koepfli, Nuno Ferrand, Raquel Godinho

**Affiliations:** 1 CIBIO/InBIO, Centro de Investigação em Biodiversidade e Recursos Genéticos, Campus de Vairão, 4485-661 Vairão, Portugal; 2 Departamento de Biologia, Faculdade de Ciências, Universidade do Porto, 4169-007 Porto, Portugal; 3 Department of Biology, University of Copenhagen, 2200 Copenhagen, Denmark; 4 Department of Zoology, Centre for Ecological Genomics and Wildlife Conservation, University of Johannesburg, 2006 Johannesburg, South Africa; 5 Smithsonian-Mason School of Conservation, Front Royal, VA 22630, USA; 6 Smithsonian Conservation Biology Institute, Center for Species Survival, National Zoological Park, Washington, DC 20008, USA

**Keywords:** 10X Genomics Chromium, Bovidae, *de novo* genome assembly, *Hippotragus equinus*, genomic diversity, roan antelope

## Abstract

Roan antelope (*Hippotragus equinus*) is the second-largest member of the Hippotraginae (Bovidae), and is widely distributed across sub-Saharan mesic woodlands. Despite being listed as "Least Concern" across its African range, population numbers are decreasing with many regional Red List statuses varying between Endangered and Locally Extinct. Although the roan antelope has become an economically-important game species in Southern Africa, the vast majority of wild populations are found only in fragmented protected areas, which is of conservation concern. Genomic information is crucial in devising optimal management plans. To this end, we report here the first *de novo* assembly and annotation of the whole-genome sequence of a male roan antelope from a captive-breeding program. Additionally, we uncover single-nucleotide variants (SNVs) through re-sequencing of five wild individuals representing five of the six described subspecies. We used 10X Genomics Chromium chemistry to produce a draft genome of 2.56 Gb consisting of 16,880 scaffolds with N50 = 8.42 Mb and a BUSCO completeness of 91.2%. The draft roan genome includes 1.1 Gbp (42.2%) repetitive sequences. *De novo* annotation identified 20,518 protein-coding genes. Genome synteny to the domestic cow showed an average identity of 92.7%. Re-sequencing of five wild individuals to an average sequencing depth of 9.8x resulted in the identification of a filtered set of 3.4x10^6^ bi-allelic SNVs. The proportion of alternative homozygous SNVs for the individuals representing different subspecies, as well as differentiation as measured by PCA, were consistent with expected divergence from the reference genome and among samples. The roan antelope genome is a valuable resource for evolutionary and population genomic questions, as well as management and conservation actions.

## Introduction

Roan antelopes (*Hippotragus equinus*) are endemic to Africa, ranging throughout sub-Saharan habitats of mesic savannah woodlands. The species comprises six recognized subspecies, namely *H. e. koba* in the northwest, *H. e. charicus* and *H. e. bakeri* across central Africa, *H. e. langheldi* in the east, and *H. e. cottoni* and *H. e. equinus* in southern Africa. These subspecies were accepted by Ansell (1972) based on geographical range, but their validity has been challenged by both nuclear and mitochondrial data (Matthee and Robinson 1999; Alpers *et al.* 2004). To fully resolve the validity of the subspecies and regional genetic differentiation, more powerful genomic tools and sampling coverage is needed. Despite being listed as “Least Concern” by the IUCN Red List of Threatened Species, the roan antelope is severely threatened by different anthropogenic pressures, including habitat loss and epizootic diseases (IUCN SSC Antelope Specialist Group 2017). Currently, most wild populations are found only within protected areas, with decreasing population trends recorded over the past decades, some resulting in local extinctions across eastern and southern Africa (East and IUCN SSC Antelope Specialist Group 1999; Chardonnet and Crosmary 2013). Over parts of its range, roan antelope has become established as an economically-important game species, leading to intensive captive breeding of animals. To this end, appropriately planned and executed translocations of wild animals are becoming an important management tool (Jansen van Vuuren *et al.* 2017), whereas the availability of reference genomes and high-resolution genomic resources add significantly to the management and conservation toolbox (Allendorf *et al.* 2010; Shafer *et al.* 2015; Dresser *et al.* 2017; Brandies *et al.* 2019).

As part of the horse-like antelopes, roan antelope is a member of the subfamily Hippotraginae, a group of bovids that consists of three genera namely *Hippotragus*, which includes the roan and the sable antelope (*H. niger*) as well as the extinct bluebuck (*H. leucophaeus*); *Addax*, a single-species genus including only the addax (*Addax nasomaculatus*); and *Oryx*, which comprises four species, namely the beisa (*O. beisa*), the scimitar-horned oryx (*O. dammah*), the gemsbok (*O. gazelle*), and the Arabian oryx (*O. leucoryx*) (Fernández and Vrba 2005; Bibi 2013). Recently, assembled genomes became available from three species within the Hippotraginae. Studies on gemsbok (Farré *et al.* 2019), the sable antelope (Koepfli *et al.* 2019), and the scimitar-horned oryx have exemplified how management and conservation actions can be mended by the use of genome-wide resources. Additionally, assembled reference genomes and inherent annotation information may also be used to address important biological questions related to adaptation to ecological conditions, both in natural and human-managed environments (Ge *et al.* 2013; Kardos *et al.* 2016; Armstrong *et al.* 2018; Martchenko *et al.* 2018).

In this study, we used the 10X Genomics linked-reads platform to sequence and assemble the first genome for roan antelope. We tested its quality, comparing the roan genome assembly to other assembled genomes generated within the Hippotraginae, Bovidae, and also to other ruminant species. We also generated and used re-sequencing data of wild individuals, representing five out of the six recognized subspecies across the roan antelope's native range, to assess general levels of intraspecific diversity.

## Materials and methods

### Sample collection, library preparation, and sequencing

For the reference genome, a tissue sample was collected from a roan antelope bull housed in the Lisbon Zoological Garden (Portugal; ID: 10954). This individual is fifth-generation captive-bred, with a genetic pool that represents an example of *ex-situ* conservation and a collaboration between several European Zoological Gardens. The animal descends from wild-caught individuals in Uganda and Zambia, which became the founders of the population in the Hannover Zoological Garden, Germany (Supplementary Figure S1). The sample was collected with a tele-biopsy dart (Vario Syringes S300V, Telinject USA, Inc.), and snap-frozen in liquid nitrogen to guarantee DNA quality. The tissue sample was processed for DNA isolation, DNA library preparation, and sequencing by the Genomic Services Laboratory at the HudsonAlpha Institute for Biotechnology (Huntsville, AL, USA). Briefly, high molecular weight DNA was isolated using a Qiagen MagAttract Kit (Qiagen, Germany). Quality was determined via pulse-field electrophoresis on a Pippin Pulse system (Sage Science, USA) using the 5-kb–430-kb protocol, and concentration determined via Qubit Fluorometric Quantitation (Thermo Fisher Scientific, USA). Approximately 1 ng of DNA was used as input for Chromium Genome library preparation (v2 chemistry), which was added onto the 10X Chromium Controller (10X Genomics Inc., USA) to create Gel Bead in-Emulsions (GEMs) from natural DNA fragments. After the run, amplified fragments within each GEM were pooled into anchored longer fragments, according to molecular barcodes, and used for subsequent steps (van Dijk *et al.* 2018). Post libraries were constructed using standard Chromium Genome i7 indexes, quantified by qPCR (Kapa Biosystems, USA), and sequenced on 2 lanes of an Illumina HiSeq X Ten system flow cell (Illumina, USA), generating 150-bp paired-end reads.

**Figure 1:**
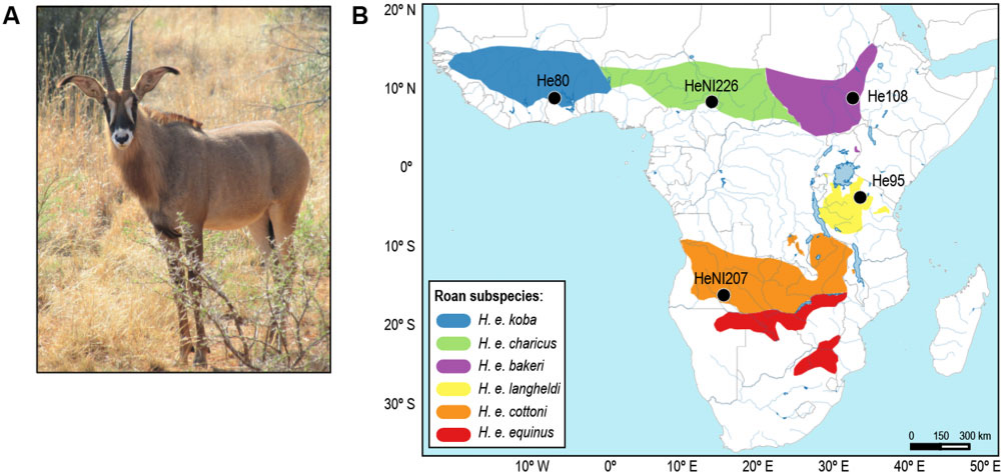
Roan antelope distribution and sampling. (A) Roan antelope (*Hippotragus equinus*). Photo by Raquel Godinho; (B) Geographic distribution of roan antelope subspecies according to Ansell (1972) and current species distribution, following IUCN SSC Antelope Specialist Group (2017). The geographic location of the five samples used in this study for whole-genome re-sequencing is represented by dark dots labelled with sample identifications (as in Table 1).

For whole-genome re-sequencing, we used five tissue samples from wild individuals, representing five out of the six currently recognized subspecies for the roan antelope (Figure 1 and Table 1). Three samples were contemporary muscle preserved in ethanol 96% from the tissue collection of the University of Copenhagen, whereas the remaining two samples were historic dried skin donated by the Powell-Cotton Museum (Kent, UK). Total genomic DNA for contemporary samples was extracted using the Qiagen DNeasy Blood and Tissue Kit (Qiagen, Germany) following the manufacturer’s instructions. For the historic samples, after re-hydration, DNA extraction was performed in a laboratory dedicated to low-quality DNA following the protocol of Dabney *et al.* (2013). Negative controls were used throughout both DNA extraction processes to monitor for potential contamination. DNA of contemporary samples was sheared by sonication and fragments of ca. 350 bp were selected using AMPure XP bead clean-up protocol (Beckman Coulter, USA). For historic samples, no fragment size-selection was performed. Historic DNA was incubated with USER (Uracil-Specific Excision Reagent) enzyme (NEB, New England Biolabs, UK) for uracil excision. Double-stranded DNA library preparation followed Meyer and Kircher (2010) and Kircher *et al.* (2012) protocols for dual-indexing. Amplification and purification were done following Dabney and Meyer (2012), after determining by qPCR the number of amplification cycles that minimizes hydrolytic damage (Swillens *et al.* 2004; Stiller *et al.* 2006) (Table 1). Amplified libraries from both contemporary and historic samples were cleaned using MinElute PCR purification kit (Qiagen, Germany), quantified by Kapa Library Quantification Kit (Roche Sequencing and Life Science, USA), and pooled at equimolar amounts. Pooled sequencing was performed on a HiSeq 4000 platform (Illumina, USA) using the 150-bp paired-end sequencing protocol.

**Table 1:** Roan antelope native samples used for whole*-*genome re-sequencing

Sample ID	Original label ID	Subspecies	Year	Sex	Locality	N PCR cycles	Mapping (%)	Mean coverage
He80	7324^a^	*H. e. koba*	1998	F	Kablima, Ghana	8	99.8	5.2x
HeNI226	NN.252^b^	*H. e. charicus*	1925	M	Nana Barya Reserve, CAR	9	99.7	5.9x
He108	9293^a^	*H. e. bakeri*	―	M	Akobe septum, Ethiopia	8	99.8	11.7x
He95	8250^a^	*H. e. langheldi*	1997	M	Maswa Game Reserve, Tanzania	10	99.7	12.2x
HeNI207	SWA.74^b^	*H. e. cottoni*	1937	F	Cubango river, Angola	7	99.7	14.1x

a Contemporary samples from tissue collection of the University of Copenhagen, Department of Biology (responsible: H.R. Siegismund).

b Historic samples donated by the Powell-Cotton Museum, Kent, UK; CAR—Central African Republic.

### Genome assembly and completeness

Total raw-data were assembled using the 10X Genomics software Supernova v.2.1.1 (Weisenfeld *et al.* 2017). We used ca. 1.2 Gbp randomly selected reads to obtain a raw read coverage of 56x, as recommended by 10X Genomics. Subsequent fasta files were generated using Supernova mkoutput, with a kmer junction of k = 48, a minimum contig size of 1,000 bp, and using the raw style, where all edges are represented by a fasta record. Raw style was compared to two additional style outputs, where branches are selected according to coverage, gaps are merged with subsequent sequences, and no reverse complement sequences are represented: (1) megabubble style, where each arm is represented by a fasta record, and (2) pseudohap style, which extracts a single pseudohaplotype per scaffold, choosing arbitrarily between maternal and paternal alleles. Duplicated scaffolds were removed with GenomeTools sequniq v.1.6.1 (Gremme *et al.* 2013).

Assembly completeness was assessed in Benchmarking Universal Single-Copy Orthologs (BUSCO) v.3.0.2 (Simão *et al.* 2015), using the Mammalia OrthoDB v9 gene set (Zdobnov *et al.* 2017) containing 4,104 genes. By comparing BUSCO results between different output assembly styles, we found that the pseudohaplotype fasta style had the highest completeness (Supplementary Figure S2) Therefore, only this assembly was retained for further analyses.

### Genome annotation and synteny

To analyze the repeat content of the roan antelope genome, we generated a *de novo* repeat library from the assembly using RepeatModeler v.2.0.1 (Zeng *et al.* 2018) which integrates both RECON v.1.08 (Bao and Eddy 2002) and RepeatScout v.1.0.6 (Price *et al.* 2005) to predominantly find transposable elements. *De novo* generated interspersed repeats were classified using the integrated script in RepeatClassifier (Zeng *et al.* 2018). Based on this custom library, the roan assembly was screened for repetitive elements using RepeatMasker v.4.0.9 (Smit *et al.* 2015) with the NCBI-RMBlast v.2.6.0+ search engine, including interspersed genomic repeats and low-complexity sequences. We used the -s option to increase sensitivity and -xsmall to produce a soft-masked output, with repeat regions indicated in lower case letters.

We used Augustus v.3.3.2 (Stanke *et al.* 2008) to identify candidate protein-coding genes in the masked-assembled genome. The software was launched disabling annotation of untranslated regions (–UTR=off), using the masked sequence as evidence against exons (–softmasking = 1) and applying the human gene model for gene prediction (–species=human). Candidate genes were translated into protein sequences using the incorporated Augustus script getAnnoFasta and filtered using eggNOG-mapper (Huerta-Cepas *et al.* 2017). Annotation quality was assessed based on eggNOG v.4.5 orthology data (Huerta-Cepas *et al.* 2016), with a minimum query cover of 50% and an e-value cut-off of 1x10^-4^. Finally, annotation quality of the roan genome was compared with two other Hippotraginae (sable antelope and scimitar-horned oryx) and the domestic cow assemblies for a set of common metrics using GenomeQC (Manchanda *et al.* 2020).

Roan genome assembly was aligned to the domestic cow genome (BosTau9—GenBank accession number: GCA_002263795.2; Zimin *et al.* 2009) using LAST v.0.874 (Kiełbasa *et al.* 2011). The domestic cow genome was first prepared for alignment using the lastdb command option, and the alignment was run using lastal and last-split commands. Then, we used the maf-swap incorporated script to change the order of the sequences into the resulting MAF-format alignment to obtain the best pairwise synteny blocks between genomes. Ordered scaffolds above 10 Kbp were used to visualize genome synteny with Circos v.0.69.6 (Krzywinski *et al.* 2009).

### Re-sequencing alignment

Read data from the five re-sequenced wild individuals were aligned to the roan antelope genome assembly using BWA-mem (Li 2013) with default settings, following adapter-trimming with Trimmomatic v.036 (Bolger *et al.* 2014) and retaining only reads > 50 bp. Duplicated reads were marked with Picard v.2.21.4 (http://broadinstitute.github.io/picard/) and local realignment around indels was improved using GATK v.3.8 IndelRealigner (McKenna *et al.* 2010). Final mapping quality was assessed with QualiMap v.2.2.2 (Okonechnikov *et al.* 2016).

### SNV calling and filtering

Filtered alignments were used in GATK v.3.8 HaplotypeCaller (van der Auwera *et al.* 2013) to separately call potential single-nucleotide variants (SNVs) for each of the five individuals. GATK GenotypeGVCFs was then used for joint genotyping based on individual genomicVCFs. Joint genotyping on cohorts improves variant calling by preventing base uncertainty errors. Using both BCFtools v.1.9 (Li *et al.* 2009) and VCFtools v.0.1.16 (Danecek *et al.* 2011), variant data were filtered to retain (1) only bi-allelic SNVs, (2) SNVs without missing data, (3) no indels, (4) SNVs covered by more than 5 reads, and (5) SNVs with a quality score > 50. The final set of filtered SNVs was used for principal component analysis, with the gdsfmt v.1.8.3 and SNPRelate v.1.6.4 R packages (Zheng *et al.* 2012).

### Data availability

Genomic resources of *H. equinus* (whole-genome assembly, Illumina paired-end reads from five individuals and samples) have been deposited at NCBI databases under BioProject PRJNA682311. The whole-genome of *H. equinus* at DDBJ/ENA/GenBank is under the accession JAEFBM000000000. The version described in this paper is version JAEFBM010000000. Supplementary material is available at figshare: https://doi.org/10.25387/g3.13378799.

## Results and discussion

### Genome assembly and completeness

The genome sequencing of roan individual ID: 10954 using 10X Genomics Chromium generated approximately 1.2x10^9^ paired-end reads (Supplementary Table S1). The raw and effective (*i.e.* the number of reads retained after filtering) read coverage recommended by 10X Genomics is 56x and 42x, respectively. The assembly summary statistics confirm the obtained values as close to the recommended ones (60.7x raw and 39.7x effective coverage; Supplementary Table S1). This genome has a contig N50 of 239.6 Kb, assembled into 16,880 scaffolds with an N50 of 8.42 Mb (Supplementary Table S1). Overall assembly quality is reflected in the high number of scaffolds (>2,200) more than 10 Kb in length and in the low percentage (4.1%) of missing bases from such scaffolds (Supplementary Table S1). Total genome assembly size was 2.56 Gb, which is close to the genome size estimated from the *k-mer* distribution of 3.01 Gb (Supplementary Table S1). The Supernova v.2.1.1 software estimated a genome-wide G + C content of 41.9%, and an overall heterozygosity of 0.0015 (one heterozygous position every 636 bp).

The assembly size and the G + C content of our roan antelope genome is comparable to other available genomes for Hippotraginae, as well as to genomes of other members of the Bovidae family and other ruminant species. For example, the assembly genome size for the European bison and the sable antelope are 2.58 Gb and 2.60 Gb, respectively (Wang *et al.* 2017; Koepfli *et al.* 2019), whereas estimates of G + C content for the sable antelope and the scimitar-horned oryx are 41.8% (Koepfli *et al.* 2019; Humble *et al.* 2020). However, the 10X Genomics Chromium library system implemented for the roan genome assembly allowed us to considerably improve the scaffold N50 (8.42 Mb) in comparison with other strategies used for the sable antelope (4.59 Mb; Koepfli *et al.* 2019), the blue wildebeest (3.5 Mb; Chen *et al.* 2019), the Tibetan antelope (2.76 Mb; Ge *et al.* 2013), or for the African buffalo (2.32 Mb; Chen *et al.* 2019). Such improvements probably reflect the unique 10X Genomics library preparation protocol, which was specifically created to obtain long-range anchored information (van Dijk *et al.* 2018). The mean heterozygosity of 0.0015 was in line with estimates within mammal species and is higher than the value observed for Hippotraginae of critical conservation concern (scimitar-horned oryx; Humble *et al.* 2020).

BUSCO evaluation of gene completeness retrieved 91.2% complete genes (3,743 out of 4,104 genes; Table 2). This value is similar to the one observed for the Thomson's gazelle (91.1%) by Chen *et al.* (2019), but lower than the values retrieved for the gemsbok (above 92%) and the sable antelope (94.8%) by Farré *et al.* (2019) and Koepfli *et al.* (2019), respectively.

**Table 2:** Assembly statistics based on gene completeness scores by BUSCO v.3.0.2 for the whole-genome assembly of roan antelope

BUSCOs	Total number	Percentage
Complete	3,743	91.2
Complete and single-copy	3,677	89.6
Complete and duplicated	66	1.6
Fragmented	194	4.7
Missing	167	4.1

BUSCO scores using the Mammalia OrthoDB v9 gene set, containing 4,104 genes.

### Genome annotation and synteny

An estimated 42.2% (about 1.1 Gbp) of the roan genome was composed of repetitive sequences, based on short- and long-interspersed nuclear elements (SINEs and LINEs), long-interspersed retrotransposons (LTR), DNA elements, small RNAs, and simple and low complexity tandem repeats (Table 3). LINEs were the most common repetitive element, representing 30.4% of the overall content, followed by LTR elements, which represented 4.0%. We also obtained a low percentage of unclassified repeats (2.5%) that did not correspond to any familiar element in the RepeatModeler specific library. Transposable elements exist in elemental families and comprise a significant fraction of eukaryotic genomes (Biscotti *et al.* 2015). Each family is derived from the multiplication of a particular element in the genome of a common ancestor and is thus a proxy for shared evolutionary history across species (Schibler *et al.* 2006; Platt *et al.* 2018; Qi *et al.* 2018). Thus, a similar percentage of the repetitive content among assemblies of roan antelope and other members of its evolutionary lineage were expected (*e.g.*, Ou *et al.* 2018). Accordingly, the 42.2% of repetitive sequences observed for the roan assembly is consistent with the genome assemblies of other Hippotraginae, such as the sable antelope (46.97%), the gemsbok (41.27%), and the scimitar-horned oryx (47.8%) (Farré *et al.* 2019; Koepfli *et al.* 2019; Humble *et al.* 2020), and also other bovids as the domestic cow (45.28%) and the European bison (47.3%) (Zimin *et al.* 2009; Wang *et al.* 2017). Additionally, long-interspersed repeats (LINEs) were the most common element found in our assembly, which is also in agreement with general knowledge for the bovine genome (Adelson *et al.* 2009). However, identification of transposable elements can be achieved through numerous available tools, and discrepancies observed across different genomes may be related to genome assembly quality and different challenges during the identification process (Saha *et al.* 2008; Makałowski *et al.* 2019).

**Table 3:** Summary of the repetitive content found in roan antelope genome assembly, using RepeatMasker

	Number	Length occupied (bp)	Percent masked
SINEs	222,376	27,885,908	1.1
LINEs	2,731,480	799,087,834	30.4
LTR elements	496,668	106,731,053	4.0
DNA elements	377,402	54,829,227	2.1
Unclassified	413,329	65,638,505	2.5
Total interspersed repeats		1,054,172,527	40.1
Small RNA	298,364	38,764,415	1.4
Simple repeats	470,855	18,232,272	0.6
Low complexity	80,149	3,946,798	0.1
Total repeats		1,106,510,938	42.2

*De novo* genome annotation with Augustus identified a total of 30,622 candidate protein-coding genes (PCGs), of which 20,518 matched common gene names, after filtering with eggNOG-mapper. Direct comparisons of these values with other assemblies could be biased, since annotation and the final number of estimated PCGs can be influenced by the assembly method and quality (Florea *et al.* 2011). We avoided this possible bias using the recently available web framework GenomeQC that enables comparison across multiple assemblies and assembly types. The final number of PCGs estimated for our roan assembly is consistent with estimates for the domestic cow (30,589; Zimin *et al.* 2009), and slightly higher than those estimated for both the sable antelope (23,846) and the scimitar-horned oryx (28,559) genome annotations (Koepfli *et al.* 2019; Humble *et al.* 2020). Such estimates can be used as proxy for the quality of roan antelope genome (Supplementary Table S2).

**Figure 2:**
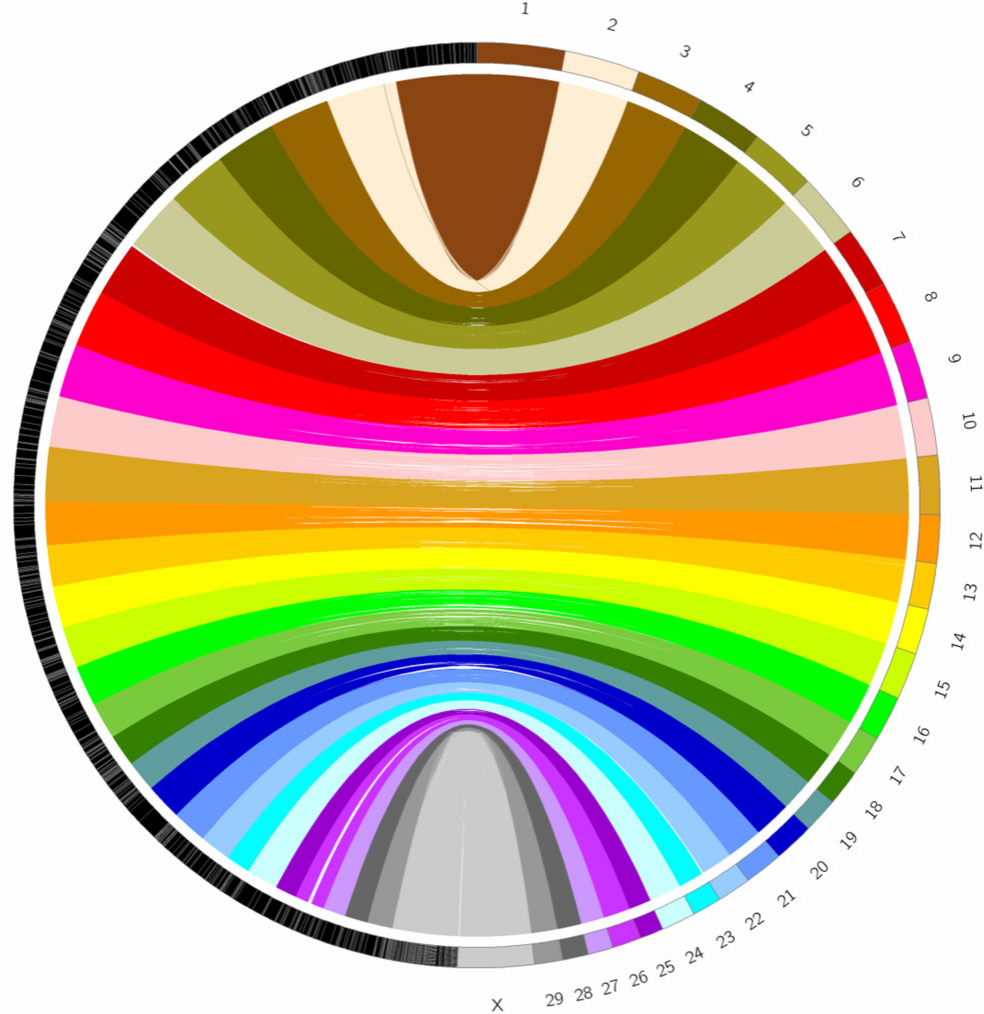
Synteny blocks between roan antelope and the domestic cow genomes. Synteny between roan scaffolds larger than 10 Kb and the domestic cow chromosomes (29 autosomes and the X). Scaffolds were ordered to obtain the best pairwise alignment blocks between the two genomes which are visualized using colored lines.

Roan scaffolds alignment against the domestic cow reference genome resulted in 85.6% synteny alignment (ca. 2.25 Gbp), with an average identity of 92.7%. Long stretches of synteny blocks covered more than 95% of each of the 29 bovine autosomal chromosomes, as well as the X chromosome (Figure 2; Supplementary Tables S3 and S4).

### Intraspecific diversity

**Figure 3:**
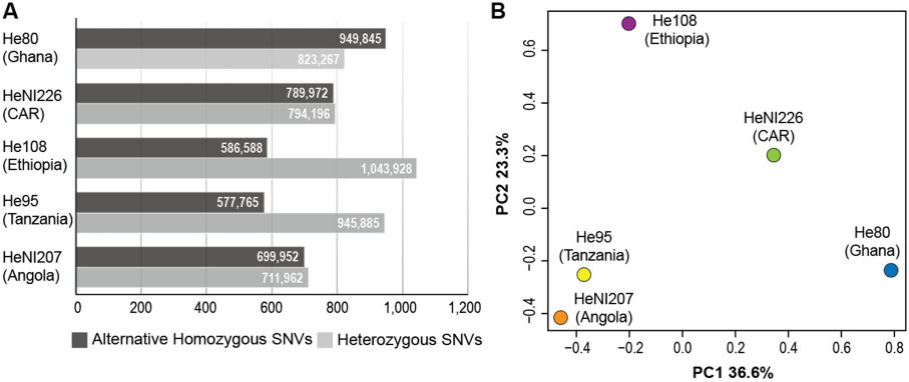
Intraspecific variation in roan antelope. (A) Bar chart comparing the number of quality-filtered alternative homozygous and heterozygous single-nucleotide variants (millions of SNVs) among the five re-sequenced wild individuals. (B) Plot of principal component analysis (PCA) for the same individuals. PC1 and PC2 show the first and second axes with corresponding percentage of explained variance. Circles identify each individual and colors correspond to the respective subspecies, following Figure 1.

Alignment of the five re-sequenced wild individuals to the roan antelope genome assembly resulted in 99.7% and 99.8% of mapped reads (Table 1). The average depth of coverage was 9.8x, ranging between 5.2x and 14.1x for Ghana and Angolan individuals, respectively (Table 1). Our intraspecific evaluation of the roan antelope genome allows the first assessment of genomic diversity patterns of the species across its native distribution range. From the alignment of the re-sequenced individuals, we found a total of 21,127,946 SNVs. Among these, 20,896,201 were bi-allelic which, after quality filtering, resulted in a final set of 3,401,741 SNVs across the five roan individuals. Overall transition/transversion ratio across SNVs was 2.07 (2,294,647/1,107,094). The number of alternative homozygous SNVs varied between 949,845 and 577,765 SNVs for individuals from Ghana and Tanzania, respectively. The number of heterozygous SNVs ranged from 1,043,928 in He108 from Ethiopia to 711,962 in HeNI207 from Angola (Figure 3A). Results for historic samples did not show any obvious bias for low coverage, reduced alignment efficiency or low number of heterozygous SNVs, which can be an important source of errors for degraded and chemically modified DNA samples (Rowe *et al.* 2011; Ewart *et al.* 2019). However, sampling size in our study hampers further interpretations.

The principal component analysis provided a visualization of genetic distances among re-sequenced individuals, which correlates with their geographical distribution (Figure 3B). The two first axes explain 60% of the total variance observed among the genome of these individuals. The first axis (PC1) explains 36.6% of the variance and separates representatives in the northern distribution of the range, namely from Ghana and Central African Republic, from those in the central and southern range of the species, namely Ethiopia, Tanzania, and Angola. The second axis (PC2), with an explained variance of 23.3%, retrieved the individual dispersion along the west-east geographical axis. Therefore, levels of intraspecific differentiation translate into a clear separation between north-south and west-east geographical axes. Although based on only a few individuals, this result supports the currently recognized subspecies and previous results on roan antelope population genetics, including the two proposed Evolutionary Significant Units (ESUs) (Ansell 1972; Alpers *et al.* 2004). Interestingly, we found a higher number of heterozygous SNVs for Ethiopian (∼1044) and Tanzanian (∼946) individuals among the five wild individuals, indicative of a higher genetic diversity. Increased intraspecific genetic diversity may be, among other factors, a signature of the presence of a contact zone, following population diversification in isolation and range expansions (Hewitt 2011). Roan antelope populations in Ethiopia and Tanzania are located in a possible contact zone between the proposed ESUs, which would extend towards west and south of those regions, respectively. Additionally, the higher proportion of reference alleles in these two individuals also indicates a closer genetic similarity to the assembled genome. This may also be explained by the evidence that the sample used to build the genome assembly is from a five-generation captive-bred individual with a maternal ancestry of wild-caught individuals from Uganda and Zambia (Supplementary Figure S1). Both countries are geographically closer to Ethiopia and Tanzania, and therefore, higher genetic similarity to the reference genome may reflect geographical proximity of these populations. Studying genome-wide diversity constitutes an important application of SNVs towards species management in both *ex-* and *in-situ* programs, as it is being applied for the sable antelope (Gooley *et al.* 2020).

### Future prospects

Non-model mammal species are difficult to sample in the wild due to cost and/or logistical constraints (Etherington *et al.* 2020). *Ex-situ* management programs, such as the ones carried out by zoological gardens, can become important donors of unique genomic resources (notably fresh tissue or non-invasive samples for DNA) for threatened and non-threatened species (Clarke 2009; Norman *et al.* 2019). From a sample collected at the Lisbon Zoological Garden, we successfully assembled the first genome for the roan antelope, one of the most iconic dwellers of the African savannah. This draft genome assembly represents a valuable genomic resource that may provide input, among others, on phylogenetic relationships, demographic history, and evolution of adaptive traits, such as headgear, multichambered stomach, and adaptation to extreme environments (Elsik *et al.* 2009; Reese *et al.* 2010; Bao *et al.* 2019; Chen *et al.* 2019). Across the three genera that comprise Hippotraginae, both *Oryx* and *Addax* species are arid-adapted, whereas *Hippotragus* species dwell in more mesic savannah habitats. The adaptation to different environments is an example of a future research topic leading to important biological discoveries among this subfamily. Furthermore, the availability of the roan antelope’s reference genome and the additional genomic resources included in this work may prove highly valuable on management and conservation decisions (Dresser *et al.* 2017; Supple and Shapiro 2018; Brandies *et al.* 2019). Improvements to the roan’s draft genome can be accomplished by proximity ligation sequencing (Hi-C) to generate chromosome-length scaffolds (Dudchenko *et al.* 2018).
